# The Contribution of Cognitive Control Networks in Word Selection Processing in Parkinson’s Disease: Novel Insights from a Functional Connectivity Study

**DOI:** 10.3390/brainsci14090913

**Published:** 2024-09-11

**Authors:** Sonia Di Tella, Matteo De Marco, Isabella Anzuino, Davide Quaranta, Francesca Baglio, Maria Caterina Silveri

**Affiliations:** 1 Department of Psychology, Catholic University of the Sacred Heart, 20123 Milan, Italy; isabella.anzuino@unicatt.it (I.A.); davide.quaranta@unicatt.it (D.Q.); mariacaterina.silveri@unicatt.it (M.C.S.); 2 Department of Life Sciences, Brunel University London, Uxbridge UB8 3PH, UK; matteo.demarco@brunel.ac.uk; 3 Department of Neuroscience, Catholic University of the Sacred Heart, 00168 Rome, Italy; 4 Neurology Unit, Fondazione Policlinico Universitario “A. Gemelli” IRCCS, 00168 Rome, Italy; 5 IRCCS Fondazione Don Carlo Gnocchi ONLUS, 20148 Milan, Italy; fbaglio@dongnocchi.it

**Keywords:** Parkinson’s disease, word selection, word production, resting-state networks, functional MRI, cognitive large-scale networks

## Abstract

Parkinson’s disease (PD) patients are impaired in word production when the word has to be selected among competing alternatives requiring higher attentional resources. In PD, word selection processes are correlated with the structural integrity of the inferior frontal gyrus, which is critical for response selection, and the uncinate fasciculus, which is necessary for processing lexical information. In early PD, we investigated the role of the main cognitive large-scale networks, namely the salience network (SN), the central executive networks (CENs), and the default mode network (DMN), in word selection. Eighteen PD patients and sixteen healthy controls were required to derive nouns from verbs or generate verbs from nouns. Participants also underwent a resting-state functional MRI. Functional connectivity (FC) was examined using independent component analysis. Functional seeds for the SN, CENs, and DMN were defined as spheres, centered at the local activation maximum. Correlations were calculated between the FC of each functional seed and word production. A significant association between SN connectivity and task performance and, with less evidence, between CEN connectivity and the task requiring selection among a larger number of competitors, emerged in the PD group. These findings suggest the involvement of the SN and CEN in word selection in early PD, supporting the hypothesis of impaired executive control.

## 1. Introduction

Parkinson’s disease (PD) is the second-most common neurodegenerative disorder and is characterized by a progressive neuronal loss in the substantia nigra pars compacta, which causes striatal dopamine deficiency. Although still considered a paradigmatic movement disorder, PD is accompanied by a broad spectrum of non-motor symptoms, such as disorders of mood and affect, cognitive impairments, alterations in emotional processing, disturbances of sleep–wake cycle regulation, and autonomic dysfunction [1,2,3,4,5,6]. Non-motor symptoms can be present from early stages of the disease, sometimes even before the appearance of classical motor features, likely in relation to nigrostriatal dopaminergic changes [7] and/or to dysfunctions of other neurotransmitter systems; other non-motor symptoms can develop later, in some cases in relation to dopaminergic medication. The loss of dopaminergic neurons in the substantia nigra contributes to motor and non-motor problems of the disease as a consequence of dysfunctions in cortico-striatal-thalamic-cortical circuitries. Basal ganglia not only are connected to motor cortical areas, but also have connections with a wide range of non-motor areas of the cortex [8].

Considering the cognitive profile and, in more detail, language abilities, when attentional resources are reduced (such as in PD), processing words denoting actions (i.e., verbs) is more difficult than processing words denoting objects (i.e., nouns) because verb-forms must be selected from a larger pool of word-forms (competitors) which share the same verb-root, and thus the set of possible verbs is larger compared to nouns [9,10]. However, in an experimental paradigm based on word morphology, designed ad hoc to make noun choice more difficult than verb choice (and to maintain the number of alternatives to choose the correct response from under control), verb production was easier, i.e., more accurate and faster, than noun production in a population of PD (for instance, if the noun “cammino” [walk] must be derived from the verb “camminare” [to walk], the choice has to be made among six possible nouns (“cammino” [walk], “camminata” [walk], “camminamento” [route], camminante [walking], camminatrice [walker, female], camminatore [walker, male]); however, when the verb base “camminare” [to walk] must be generated from the derived noun “cammino” [walk], only one option effectively exists). The results confirmed that lexical production is conditioned, at least in part, by the difficulty of the task and not by the grammatical class (noun or verb) of the word. In this context, PD performance was compatible with the presence of a dysexecutive syndrome and reduced attentional resources during the process of lexical selection [11].

In previous studies, using the same experimental paradigm described above, we obtained evidence that the word selection process that is more demanding of attentional resources is correlated with cortical thickness of the left inferior frontal gyrus (IFG) [12]. Moreover, in another study [13] using the Diffusion Tensor Imaging (DTI) technique in a PD sample, we demonstrated that microstructural changes in the uncinate fasciculus, the white matter bundle that connects the IFG to the anterior temporal lobe (ATL), which is recognized as a critical multimodal semantic hub, correlated with the performances in word selection processing.

The investigation of word selection processes in PD might be supported by resting-state functional magnetic resonance imaging (rs-fMRI), which detects changes in the blood oxygen level-dependent (BOLD) signal and has been used to map the brain’s functional organization based on the intercorrelations among regions (functional connectivity, FC). Using this technique, a scaffold of connected brain regions that facilitates signaling along preferred pathways at the service of specific functions has been identified and recognized to exert coordinated effects to sustain a range of cognitive functions [14]. In particular, the three core neurocognitive networks—the default mode network (DMN), the fronto-parietal central executive network (right and left CENs), and the salience network (SN)—are thought to interact dynamically to influence cognitive performance [15,16,17]. The DMN, centered in the posterior cingulate cortex, the medial prefrontal cortex, and the angular gyrus, is a network active when a subject is awake and at rest, it is an “intrinsic” system, specialized in internally oriented cognitive processes such as daydreaming, reminiscing, and future planning, while the arrival of attention-grabbing stimuli from the outside inhibits it [18,19,20,21]. The CEN, mainly expressed in the dorsolateral prefrontal cortex and the posterior parietal cortex, supports functions that require significant cognitive activation, with a strategic connotation, such as solving complex problems and the ability to make decisions [15,16]. Paralimbic structures, which are part of the SN, are critical for cognitive control, most prominently the dorsal anterior cingulate cortex, the left and right anterior insula, and the adjacent IFG [16], coactivate in response to varied forms of salience; in other words, in the detection of potentially relevant stimuli. The anterior insula acts as a “cortical outflow hub” coordinating changes in activity across multiple brain regions [17,22,23,24] and in switching activation between the DMN and CEN [22]. The interaction between the SN and DMN is crucial in the control of attention required for the most demanding cognitive tasks. In healthy young adults, greater anti-correlation (negative correlation) between the SN and DMN has been associated with more efficient cognitive control [25]. Failures in deactivation of DMN have been associated with reduced sustained attention [26], which is observed across many neurological conditions [27].

While a lot of data are available on the brain circuits associated with motor impairments in PD, less is known about the structure and function of brain networks that contribute to cognition. Disruptions in the DMN, CEN, and SN have been reported in PD and found to be associated with cognitive performance [28,29,30,31,32,33,34,35]. A recent meta-analysis [31] documented reduced FC predominantly in the DMN and CENs when PD patients with cognitive impairment were compared with HC. However, DMN alteration can precede objective cognitive impairment in PD. Tessitore et al. [32] found decreased DMN connectivity even in a cohort of cognitively unimpaired PD patients, and reported positive correlations between DMN connectivity and cognitive performance in tests of memory and visuospatial functioning [32]. Moreover, aberrant CEN connectivity seems to have a critical role in determining the “dysexecutive syndrome” in PD, the typical cognitive profile observed in PD patients due to fronto-stratial disconnection [16], whereas the integrity of this network is associated with preservation of cognitive profile [33,34,35]. Reduced FC of CENs has been associated with worse cognition in PD [36]. Disruptions in the SN have also been reported in PD. In fact, this network becomes dysfunctional in PD, as its key nodes, the anterior insula and the dorsal anterior cingulate cortex, become direct targets of PD pathology [29,30], being vulnerable regions to alpha-synuclein deposition according to Braak’s staging hypothesis [37]. In PD, it has been proposed that as the SN becomes dysfunctional, it is no longer able to suppress DMN activity effectively [23,38]. Furthermore, a recent study [39] investigating the dynamic relationship between FC of canonical cortical and subcortical networks, and cognitive dysfunction in PD, showed that cognitive impairment assessed by a screening test such as the MoCA test is associated with reduced FC within CEN and reduced FC between CEN and DMN, CEN and SN, and SN and subcortical basal ganglia networks.

The novelty of our study is the use of a task specifically devised to explore cognitive control during the word selection process in PD, in order to correlate performance to FC of large brain networks. In particular, we investigated in early PD without severe cognitive impairment if word selection processing might be modulated by the activity of major neurocognitive functional networks. Our hypothesis is that CEN plays a specific role during highly demanding lexical production processes in which the difficulty of the task depends on the number of alternatives among which the choice is made. We also expected an involvement of the SN to the extent that it predisposes the activation of the CEN, by inhibiting the activity of the DMN. A correlation between performance and FC of major cognitive control networks (i.e., the SN and CEN) would confirm the involvement of these large brain networks in cognition and would support the hypothesis that word selection processes in PD may be interpreted in the context of dysexecutive syndrome.

## 2. Materials and Methods

### 2.1. Participants

Eighteen PD participants and sixteen matched healthy controls (HCs) were enrolled in the study. All participants were right-handed according to the Edinburgh Handedness Inventory [40]. PD patients were selected from those referred to the IRCCS Don Carlo Gnocchi Foundation ONLUS—Neurological Unit (Milan, Italy) following the subsequent inclusion criteria: (1) diagnosis of probable PD according to the United Kingdom Parkinson’s Disease Society Brain Bank [41]; (2) nigro-striatal dopaminergic neurodegeneration as detected by DaTSCAN imaging; (3) mild to moderate stage of the disease on the Modified Hoehn and Yahr (H&Y) Scale (score between 1 and 2.5) [42]; (4) education ≥ 8 years; (5) Italian native speaker; (6) absence of cognitive impairment as assessed by the Montreal Cognitive Assessment Test (MoCA) (score greater than or equal to 17.54) [43]; (7) stable therapy with L-Dopa or dopamine agonists, catechol-O-methyltransferase inhibitors, monoamine oxidase inhibitors, and anticholinergic drugs; and (8) absence of on–off fluctuations and dyskinesias due to medication. Patients were not included in the study if they presented clinical signs fulfilling criteria for other neurological disorders, comprising possible atypical parkinsonisms; secondary or iatrogenic parkinsonism; neuropsychiatric disorders besides PD diagnosis; or claustrophobia. The neurological examination of PD included the H&Y scale [42] and the Unified Parkinson’s Disease Rating Scale (UPDRS)—motor part III [44]. Patients also underwent a comprehensive neuropsychological assessment of global cognitive efficiency (Mini Mental State Examination, MMSE; and MoCA), linguistic functions (object and action oral naming; phonological fluency; semantic fluency), verbal and spatial memory (Immediate and Delayed Recall of 15 words; Free and Cued Selective Reminding Test—FCSRT; Rey–Osterrieth figure recall), verbal and spatial short term memory (verbal span and Corsi’s test), intelligence (Raven Colored Matrices), visuo-constructional and praxis abilities (Rey–Osterrieth figure copy), and attention and executive functions (Trail making test, TMT part A, B and B-A; Attentional Matrices; Stroop test; Modified Wisconsin Card Sorting test, M-WCST), lasting about 2 h, over two sessions. Sixteen age–sex–education matched HCs were included in the study as a control group. The neuropsychological evaluation of HCs was less extensive and included the MMSE, MoCA, phonological and semantic fluency, and TMT.

### 2.2. Experimental Paradigm: Languange Tasks

The experimental paradigm consisted of word production tasks described in previously published studies (see [11,12], for details). For each task, the input stimuli were provided in a random order in two sets, i.e., two sets of 72 verbs (144 items for the derivation task) and two sets of 72 nouns (144 items for the generation task). More precisely, during the verb-from-noun (V_from_N) generation task, participants were instructed to turn the noun into the corresponding infinitive form of the verb. For instance, if the noun “sentimento” [feeling] was presented on the screen, they were requested to say the verb “sentire” [to feel] out loud as quickly as possible. In the noun-from-verb (N_from_V) derivation task, participants were asked to turn the verb into the corresponding noun. For instance, if the verb “partire” [to depart] was presented on screen, they were requested to say the noun “partenza” [departure] out loud as quickly as possible. A training session preceded each set, where participants were instructed to say the expected target out loud as quickly and accurately as possible. During the training session, the examiner could provide feedback on the accuracy of participants’ responses. Once the actual task had started, participants did not receive any feedback. The whole experimental session lasted about 60 min with a break after each set.

Words were controlled for psycholinguistic parameters (see [45]). A relevant aspect to note is that the number of alternatives among which the subject had to select his/her response varied across the two sets of stimuli. To test the experimental hypothesis as described in previously published studies (see [11,12], for details), this variable was estimated considering the number of word-types that share the root with the input word and are expected to be involved in processing the response. These word-types are annotated in the corpus of written Italian by Bertinetto et al. [46] (CoLFIS, http://linguistica.sns.it/CoLFIS/Home.htm accessed on 1 August 2024). When the verb was the input (N_from_V derivation task), the target had to be selected among several alternatives (range: 1–8; M = 3.1; SD = 1.6) whereas, when the noun was the input (V_from_N generation task), there were no concurrent alternatives, but only one alternative, considering that from a noun target only one verb base could be retrieved.

Stimuli were administered using SuperLab pro Software (version 2.0.4) (Cedrus, Phoenix, Arizona) and presented one at time, in bolded black font and size 60, on the center of a computer. The experiment was carried out in a quiet room, with participants sitting 40 cm away from the video display. The presentation of the word “via” [start] on the screen began each set, and the appearance of the word “fine” [the end] ended each set. The trial sequence began with a blank background for a duration of 250 ms, after which a fixation point was presented for a duration of 750 ms and then the input word for a duration of 5000 ms. SuperLab pro Software can record response times (RTs), which represent the latency from the appearance of the word on display and the onset of subjects’ response. The software automatically generates an Excel worksheet in which RTs for each stimulus are reported. Response accuracy was scored manually by the examiner. The assessment of PD patients medicated with L-DOPA was completed in the “off” state, i.e., following a period of at least 12 h off antiparkinsonian medications on the day of neuropsychological and language tests.

### 2.3. MRI Acquisition Processing and Modeling

Each participant was invited to the IRCCS Fondazione Don Carlo Gnocchi ONLUS, Milan, Italy, to complete a magnetic resonance imaging (MRI) protocol consisting of structural and functional sequences. All scans were acquired on a Siemens Avanto scanner (1.5 T), equipped with a 12-channel head coil.

The following acquisitions (inclusive of technical specifications) were recorded as part of this study: (1) a 3D high-resolution magnetization-prepared rapid gradient echo (MPRAGE) T1-weighted image (repetition time (TR) = 1900 ms, echo time (TE) = 3.3 ms, inversion time (TI) = 1100 ms, matrix size = 192 × 256 × 176, resolution = 1 mm^3^ isotropic); (2) a resting-state fMRI (rs-fMRI) sequence (TR = 2570 ms, TE = 15/34/54 ms, matrix size = 64 × 64 × 31, resolution = 3.75 × 3.75 × 4.5 mm^3^, 200 volumes); and (3) a dual-echo turbo-spin echo proton-density/T2-weighted sequence [repetition time (TR) = 5550 ms, echo time (TE) = 23/103 ms, matrix size = 320 × 320 × 45, resolution 0.8 × 0.8 × 3 mm^3^]). To standardize the procedure of data acquisition, each participant was requested to lay supine, with their eyes closed, and be as motionless as possible during the entire scanning session.

All anatomical images were reviewed by a senior neuroradiologist, in order to identify the presence of potential exclusion criteria incompatible with the diagnostic categories investigated in this study, such as macroscopic brain lesions or an excessive amount of confluent white matter hyperintensities.

The analyses (i.e., preprocessing and modelling) were carried out via Statistical Parametric Mapping (SPM) 12 (Wellcome Centre for Human Neuroimaging, London, UK, www.fil.ion.ucl.ac.uk/spm/software/, accessed on 1 August 2024), running in a MATLAB R2014a (Mathworks Inc., Cambridge, UK) environment.

Preprocessing of resting-state fMRI images included the following steps: slice-timing, realignment, normalization, filtering, and smoothing. Slice-timing was carried out to synchronize each volume by registering all slices to a common temporal reference. Realignment served instead to correct the spatial position of each volume, and thus calculate volume-to-volume linear and rotational displacement. A graphic representation of the six displacement vectors (i.e., three translational, three rotational) was reviewed to flag problematic acquisitions showing motion profiles characterized by >3 mm translations or >3° rotations. Images were then normalized in the Montreal Neurological Institute (MNI) space, and the voxel dimension was set to a 2 mm isotropic resolution.

A band-pass filter was applied to all normalized images in order to discard non-neurogenic sources of signal variability (e.g., signal drifts originating from the scanner, and cardiorespiratory rhythms). The REST toolbox [47] was used to this end, and frequencies outside of the 0.01–0.1. Hz interval were filtered out. All images were finally smoothed with a 6 mm^3^ full-width at half maximum Gaussian kernel.

A group-level independent component analysis was run to estimate large-scale networks of interest [48]. This was carried out using the Group ICA Of fMRI Toolbox (GIFT) software package (v1.3i; https://trendscenter.org/software/gift/ (accessed on 1 August 2024)). This methodology analyzes observed signal variability to discover a series of latent sources (i.e., components) that are spatially independent and are either interpreted as a functional neural system (i.e., a network) or as an artefact [49]. An a priori, literature-informed number (i.e., 20) of components was specified [50] and, following a 100% agreement rate between two independent raters [S.D.T. and M.D.M.], the following six large-scale networks were selected based on their spatial features: DMN, SN, left CEN, right CEN, sensorimotor network (SMN) and visual network (VN). SMN and VN were included in the study for methodological-control purposes, i.e., SMN as a network not primarily associated with cognitive functioning, yet affected by PD and significantly associated with the severity of motor symptoms caused by the disease [51], and VN as a network neither primarily associated with cognitive functioning, nor typically affected by PD.

### 2.4. Statistical Analyses

Demographic, clinical, and neuropsychological descriptives were expressed as means and standard deviations (SD) or frequencies and percentages, as appropriate. Differences between the two diagnostic groups were tested with chi-squared (χ^2^) tests, independent samples *t*-tests, or Mann–Whitney U tests, according to the variables (categorical or continuous, normally or non-normally distributed), with a threshold of statistical significance set at *p* < 0.05.

In order to expand the analysis of fMRI data by focusing on its network core hubs, all subject-specific maps back-reconstructed by ICA (*n* = 34) were analyzed via one-sample *t*-tests to confirm the group-level regional contour of each component. Results of these one-sample *t*-tests were considered significant when surviving an uncorrected *p* < 0.00001 cluster-forming threshold, and a Family-Wise-Error (FWE)-corrected *p* < 0.05 at a cluster level.

The output of these six analyses was then inspected, and the MNI coordinate expressing the peak z-score in each resulting map was identified. A spherical region of interest (ROI; radius: 4 mm) was then constructed around each of these six coordinates via the MarsBaR toolbox for SPM [52], and this same software tool was also used to extract the six ROI time-courses from the preprocessed scan of each individual. Each vectorial ROI time-course was, in turn, averaged across all volumes to obtain six scalar indices of ROI network expression.

To test the association between neurofunctional data and language task performance, partial correlations (Pearson’s *r* coefficients) were calculated between the six indices of ROI network expression and the accuracy and lnRTs (after logarithmic transformation of RTs) of word production tasks (V_from_N, N_from_V, and overall word production, indicated as W_production). Age, sex and education were included as covariates. These correlations were run with IBM SPSS Statistics software (version 29.0.1.0).

The magnitude of correlations was interpreted as follows: |0.1–0.3| as a small association; |0.3–0.5| as an intermediate association; and |0.5 and higher| as a strong association [53]. Moreover, to adjust for multiple comparisons, the false discovery rate (FDR) correction [54] was calculated with the “p.adjust” package (https://rdrr.io/cran/POSTm/man/p.adjust.html, accessed on 1 August 2024) implemented in RStudio statistical software (version 2023.03.0), which provides adjusted *p*-values from a set of *p*-values. The statistical threshold for this correlation analysis was set at *p* < 0.05.

## 3. Results

### 3.1. Demographic, Clinical, and Neuropsychological Characterization of the Samples

HC and PD groups did not differ in age, years of education, or sex (Table 1). A significant difference was observed in global level of cognitive functioning (MoCA test, *t* (32) = −2.74, *p* = 0.010, Cohen’s *d* = 0.94), although no participant performed under the cut-off point/outer tolerance limit (15.5) or even under the inner tolerance limit (17.54) indicated by Santangelo et al. [43]. Results showed that PD patients presented an overall preserved cognitive profile, as documented by no statistically significant differences compared to HCs at the neuropsychological level, except for the TMT part A sub-test, where PD patients were slower than HCs (t (32) = 3.04, p = 0.005; Cohen’s *d* = 1.05). See Supplementary Table S1 for further details about the neuropsychological assessment. Clinically, our PD patients were classified in the initial stage according to the Modified Hoehn and Yahr (H&Y) Scale [42] and all of them showed mild disease severity to UPDRS—motor part III (minimum score 6—maximum score 41), under the cut-off point of 59 which indicates moderate/severe levels [55].

### 3.2. Resting-State Functional ROIs

**Figure 1:**
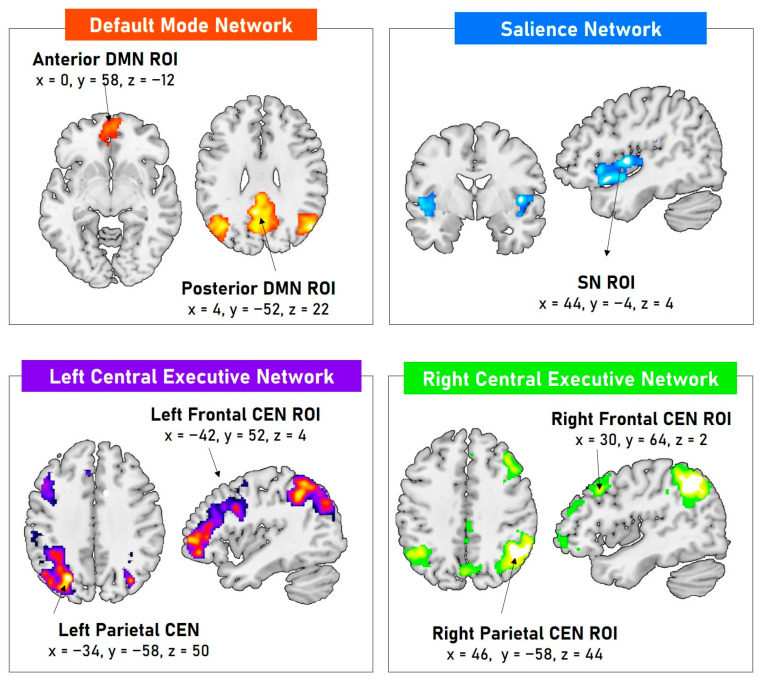
Extracted resting-state functional cognitive control network maps (one-sample *t*-tests significant at FWE corrected *p* < 0.05 at a cluster level and *p* < 0.00001 at uncorrected set level) and ROIs defined as 4 mm radius spheres in the local maxima.

The DMN was centered on the medial prefrontal cortex (anterior DMN ROI—MNI coordinates: x = 0, y = 58, z = −12) and the precuneus (posterior DMN ROI—MNI coordinates: x = 4, y = −52, z = 22); the SN was centered on the right insula (SN ROI—MNI coordinates: x = 44, y = −4, z = 4); the right CEN was centered on the middle frontal gyrus (right frontal ROI—MNI coordinates: x = 30, y = 64, z = 2) and inferior parietal lobule (right parietal ROI—MNI coordinates: x = 46, y = −58, z = 44); the left CEN was centered on middle frontal gyrus (left frontal ROI—MNI coordinates: x = −42, y = 52, z = 4) and inferior parietal lobule (right parietal ROI—MNI coordinates: x = −34, y = −58, z = 50); the SMN was centered on the precentral gyrus (SMN ROI—MNI coordinates: x = −42, y = −20, z = 58); and VN was centered on the lingual gyrus (VN ROI—MNI coordinates: x = 4, y = −66, z = 8). See Figure 1 for ROI selection.

### 3.3. Association between Language Task Performance and Resting-State Functional ROIs

Partial correlation analyses, including age, sex, and education as covariates, revealed the presence of a significant association between SN ROI connectivity and task performance in both noun and verb production and in the total number (nouns + verbs) of words produced (W_production) (accuracy: V_from_N: *r* = −0.668, *p* = 0.007; N_from_V: *r* = −0.661, *p* = 0.007; W_production: *r* = −0.747, *p* = 0.001; lnRTs: V_from_N: *r* = 0.677, *p* = 0.006; N_from_V: *r* = 0.579, *p* = 0.024; W_production: *r* = 0.653, *p* = 0.008). An association was also found between right frontal CEN ROI connectivity and latency in N_from_V production and in total number of words produced (lnRTs: N_from_V: *r* = −0.536, *p* = 0.039; W_production: *r* = −0.535, *p* = 0.040). A tendency also emerged in verb production (lnRTs: V_from_N: *r* = −0.483, *p* = 0.068).

Conversely, no significant associations were found between the FC of selected ROIs and the task scores in the HC group. Only a tendency was observed for the SN ROI connectivity and N_from_V accuracy (*r* = 0.544, *p* = 0.055), the most difficult task.

**Figure 2:**
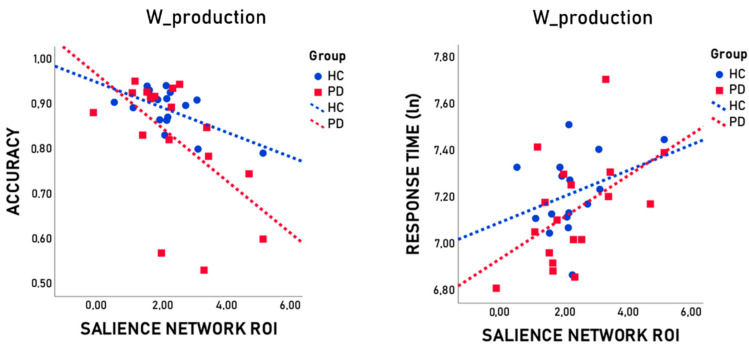
Scatterplots represent the association between SN ROI connectivity and overall task performance (W_production) expressed in terms of accuracy and response times after logarithmic transformation (ln).

It is worth noting that, after applying an FDR correction for multiple comparisons using the statistical software RStudio, only correlations between the SN ROI connectivity and task performance survived (accuracy: V_from_N: *p_FDR_* = 0.010; N_from_V: *p_FDR_ =* 0.010; W_production: *p_FDR_ =* 0.006; lnRTs: V_from_N: *p_FDR_* = 0.010; N_from_V: *p_FDR_* = 0.024; W_production: *p* = 0.010). See Table 2 and Figure 2 for further details of correlational analyses.

## 4. Discussion

We aimed to explore whether word selection processing, evaluated by an experimental paradigm that enables manipulation of task difficulty by controlling the number of alternatives among which word selection operates, is related to the FC of the main cognitive control networks in PD. In fact, the emerging network paradigm is becoming increasingly useful in understanding the neural correlates of cognition [56]. In this context, resting-state fMRI makes it possible to study the connectivity and the role of large-scale brain networks that cannot be easily captured by other existing techniques.

The present study was based on our previous findings showing that the word production deficit in PD, often reported in the literature [10,57], may be influenced by a dysexecutive syndrome [11,12,58], in relation to the difficulty of the task, determined by the number of alternatives from which to select the target, i.e., the target’s competitors.

We could document a significant inverse correlation between the accuracy of all word selection tasks (the overall accuracy and the accuracy in the production of both the noun and verb) and the FC of the SN. Furthermore, we found a significant direct correlation between the FC of the SN and longer RTs in all word selection tasks. A significant association also emerged between RTs of overall word production and of noun production (the most difficult task) and the FC of the right frontal CEN (although not surviving after multiple comparison adjustment).

No correlation was found in the HC group, although a tendency toward significance was observed between the FC of the SN and performance in noun production, i.e., the condition in which the number of alternatives from which to choose is larger.

The association found in PD between word production tasks requiring the recruitment of executive resources and the FC of the SN ROI is worthy of attention. This functional ROI is centered in the anterior insula long gyrus (according to the neuroanatomy of the insula and probabilistic atlases [59,60,61]), i.e., the “cognitive region” among the distinct subdivisions within the human insula (the other subdivisions being involved in sensorimotor, olfactory-gustatory, and socioemotional processes) (see [62] for a metanalysis of nearly 1800 functional neuroimaging experiments). The anterior insula is well recognized as a critical hub of the SN supporting executive control, considering its strong connections with the prefrontal cortex [17,63,64,65], which, in turn, is involved in higher-level cognitive control, such as planning and organization, goal-directed behavior, adaptation, and decision-making [66]. According to a network approach, during cognitive tasks the SN generally contributes to switching activation from DMN to the CEN [19] and to allocating attentional resources. There is strong evidence that functional changes in the SN are related to cognitive impairment in PD [67]. Moreover, the anterior insula is severely affected in another synuclein pathology associated with cognitive disorders, i.e., Lewy body disease, at advanced stages [68]. Given the functional importance of SN in several cognitive functions and its susceptibility to neuropathological change, the altered FC of the SN, which also includes the IFG, might be at the basis of the lexical selection difficulties documented in our patients, requiring controlled retrieval and inhibition of irrelevant competitive alternatives.

The association between RTs and the FC of the CEN (right frontal ROI), although not surviving multiple comparison corrections in the overall word production and in the most difficult task (production of nouns), is potentially relevant. In fact, RTs might be particularly sensible to the early stage of the disease, when accuracy is still relatively preserved. A recent fMRI study investigating functional interactions between brain networks and cognitive decline in a cohort of 50 PD patients showed that altered connection involving fronto-parietal networks is linked to worsening of executive functioning [69]. Accordingly, in our study, the noun-from-verb derivation task generated significant correlations with the CEN, as expected on the base of the attentional demand needed to select from many alternatives. In the presence of more competitors, the involvement of CEN emerges as a function of the difficulty of the task.

The lack of significant correlations with the FC of the DMN is not unexpected given the executive nature of the word selection task, which requires not only linguistic competence but also executive abilities in producing words that are differentially demanding of attentional resources. The lack of significant correlations between the FC of the DMN in our study is in line with the deactivation of the DMN during cognitive tasks that require attentional control [18,19,20,21].

To summarize, the findings reported in the present study seem to support the hypothesis that word selection processing in early PD might be vulnerable to the decay of executive resources typical of this pathology. The current research indicates that the SN may be generally involved in word selection processing, and this is consistent with the hypothesis that the involvement of CEN may be more specific when the tasks are particularly demanding of attentional executive resources. The current study could inform the identification of novel biomarkers (i.e., functional brain networks) of cognitive dysfunction in PD and in other neurodegenerative conditions.

Although non-motor symptoms impair quality of life just as severely as motor symptoms, resulting in severe negative social consequences [70], there are few effective treatments for these symptoms because the neural mechanisms of cognitive impairment in PD are poorly understood. This work, which supports previous studies that have highlighted the SN and CEN as critical networks for high-level cognitive functioning, could have clinical implications for nonpharmacological treatment in early stages of PD progression. In this perspective, cognitive large-scale networks represent potential targets of non-invasive treatment approaches, such as non-invasive brain stimulation, which can be used to improve quality of life and maintain autonomy in daily living. Clinicians should incorporate the management of cognitive dysfunction into a holistic treatment plan [71]. For example, during rehabilitation treatment, training selection control abilities may help PD patients to improve word production and generally their quality of life, considering the relevance of language in social interaction.

Several limitations should be considered when interpreting our results. First, most PD patients were taking dopaminergic medication. Levodopa can interfere with fMRI [72] and cerebral blood flow [73,74]. However, our sample included clinically homogeneous patients under stable pharmacological treatment; they were considered to be in the practically defined “off” state (a period of at least 12 h off antiparkinsonian medications) when they performed the language test. Studies in drug-naïve patients would be useful to control for the effects of dopaminergic drugs on functional testing. Moreover, only patients in mild to moderate stages of the disease took part in the study, whereas those with advanced PD were excluded. A higher H&Y staging means further loss of dopaminergic neurons in the nigrostriatal pathway, which may result in more complex symptoms. More research is needed to establish how PD influences word selection processing and how the connectivity of large-scale networks changes as the disease progresses. Second, the relatively small number of observations is a potential limitation of our study. Future research should include larger samples stratifying participants into cognitively impaired and demented PD patients, as demented PD patients could be ideal candidates for investigating neural networks associated with cognitive decline in PD. Third, we used a single imaging modality of investigation and selected functional ROIs based on a data-driven approach; future studies should include multimethod confirmations, integrating rs-fMRI with other functional approaches, incorporating task performance data directly in the scanner through a task-based approach, and including measures of connectivity efficiency derived from graph theory approaches. Further research could also provide evidence linking cognitive impairments in PD with inter-network FC and other measures of structural connectivity in white and gray matters. Moreover, future studies could examine connectivity between the SN and the CEN large-scale networks on the one hand, and the basal ganglia on the other, in word selection processing. The alteration of distinct subdivisions of basal ganglia loops, particularly the corticostriatal circuitry, is of great relevance in PD because dopaminergic depletion in this circuitry can profoundly alter functional brain networks and cognition [75].

Finally, our data may not contribute to the discussion about the other main hypothesis proposed in the literature to explain the typical deficit for verbs in PD, that is, the decay of conceptual representation of the motor components of the action in a pathology typically dominated by disorders of movement [57,76,77,78], according to an embodied view of cognition [79]. Future studies adopting tasks specifically devised to explore semantic processing in different attentional contexts (e.g., bottom-up as well as top-down semantic controlled retrieval) [80] might contribute to understanding the relationship between the SN, CEN, and DMN in order to also investigate the representational level of the action.

## 5. Conclusions

Our findings provide new insights into the relationship between FC of brain networks and distinctive cognitive functions like the executive control implicated in high-level language tasks.

## Figures and Tables

**Table 1:** Demographic and clinical data of HC (healthy control) and PD (Parkinson’s disease) groups of participants.

Demographical and Clinical Data	HC [n = 16]	PD [n = 18]	Group Comparison *p*-Value
Age [Mean ± *SD*] years	65.13 ± 7.53	66.83 ± 7.37	0.509 ^#^
Education [Mean ± SD]	13.56 ± 3.90	12.72 ± 4.01	0.541 ^°^
Sex M/F [n (%)]	9(56.3%)/7(43.7%)	9(50%)/9(50%)	0.716 ^§^
MoCA [Mean ± SD]	26.20 ± 2.78	23.27 ± 3.36	0.010 ^#^
UPDRS—motor part III [Mean ± SD]		21.72 ± 9.43	
Disease duration [Mean ± SD]		38.22 ± 29.55	
LEDD [Mean ± SD]		274.20 ± 208.30	

SD, standard deviation; M, male; F, female; MoCA, Montreal Cognitive Assessment Test; UPDRS—motor part III, Unified Parkinson’s Disease Rating Scale—motor part III; LEDD, levodopa equivalent dose; ^#^ independent samples *t*-test; ^°^ Mann–Whitney *U* test; ^§^ chi-squared (χ2) test.

**Table 2:** Partial correlation analysis between language task performance (accuracy and lnRT) and resting-state functional ROIs in HC (healthy control) and PD (Parkinson’s disease) groups. Pearson’s *r* correlation coefficients are reported together with *p*-values: uncorrected (*p*) and adjusted according to Benjamini and Hochberg [54] false discovery rate correction (*p_FDR_*). Significant* p*-values (*p* < 0.05) are highlighted in bold.

Partial Correlations			Accuracy			lnRTs		
Group			V_from_N	N_from_V	W_Production	V_from_N	N_from_V	W_Production
HC	Anterior DMN ROI	*r*	−0.059	0.081	0.079	0.367	0.299	0.342
		*p*	0.849	0.792	0.797	0.217	0.321	0.253
		*p_FDR_*	0.849	0.849	0.849	0.642	0.642	0.642
	Posterior DMN ROI	*r*	−0.450	0.060	−0.039	−0.041	−0.040	−0.041
		*p*	0.123	0.846	0.900	0.895	0.897	0.895
		*p_FDR_*	0.738	0.900	0.900	0.900	0.900	0.900
	SN ROI	*r*	0.117	−0.544	−0.410	0.216	0.195	0.211
		*p*	0.703	0.055	0.165	0.479	0.523	0.489
		*p_FDR_*	0.703	0.330	0.495	0.628	0.628	0.628
	Left Frontal CEN ROI	*r*	−0.359	0.204	0.071	0.124	0.021	0.073
		*p*	0.229	0.505	0.818	0.686	0.947	0.812
		*p_FDR_*	0.947	0.947	0.947	0.947	0.947	0.947
	Left Parietal CEN ROI	*r*	−0.131	0.270	0.171	−0.068	−0.233	−0.158
		*p*	0.669	0.372	0.577	0.826	0.444	0.607
		*p_FDR_*	0.802	0.802	0.802	0.826	0.802	0.802
	Right Frontal CEN ROI	*r*	0.215	0.227	0.311	0.064	0.024	0.045
		*p*	0.481	0.455	0.301	0.836	0.937	0.885
		*p_FDR_*	0.937	0.937	0.937	0.937	0.937	0.937
	Right Parietal CEN ROI	*r*	0.051	0.225	0.244	0.067	−0.129	−0.036
		*p*	0.869	0.459	0.422	0.829	0.676	0.907
		*p_FDR_*	0.907	0.907	0.907	0.907	0.907	0.907
	SMN ROI	*r*	−0.313	−0.120	−0.206	−0.475	−0.465	−0.485
		*p*	0.298	0.695	0.500	0.101	0.109	0.093
		*p_FDR_*	0.447	0.695	0.600	0.218	0.218	0.218
	VN ROI	*r*	0.226	0.183	0.172	−0.216	−0.415	−0.331
		*p*	0.458	0.549	0.575	0.478	0.159	0.270
		*p_FDR_*	0.575	0.575	0.575	0.575	0.575	0.575
PD	Anterior DMN ROI	*r*	0.002	0.467	0.339	−0.169	−0.274	−0.235
		*p*	0.995	0.079	0.216	0.548	0.323	0.399
		*p_FDR_*	0.995	0.474	0.598	0.658	0.598	0.598
	Posterior DMN ROI	*r*	−0.187	0.229	0.107	0.021	−0.082	−0.036
		*p*	0.505	0.412	0.703	0.942	0.772	0.899
		*p_FDR_*	0.942	0.942	0.942	0.942	0.942	0.942
	SN ROI	*r*	−0.668	−0.661	−0.747	0.677	0.579	0.653
		*p*	**0.007**	**0.007**	**0.001**	**0.006**	**0.024**	**0.008**
		*p_FDR_*	**0.010**	**0.010**	**0.006**	**0.010**	**0.024**	**0.010**
	Left Frontal CEN ROI	*r*	0.037	0.387	0.277	−0.209	−0.358	−0.303
		*p*	0.895	0.155	0.317	0.454	0.190	0.273
		*p_FDR_*	0.895	0.475	0.475	0.545	0.475	0.475
	Left Parietal CEN ROI	*r*	0.054	0.207	0.156	−0.392	−0.285	−0.350
		*p*	0.848	0.459	0.579	0.148	0.303	0.201
		*p_FDR_*	0.848	0.688	0.695	0.603	0.606	0.603
	Right Frontal CEN ROI	*r*	0.144	0.293	0.264	−0.483	−0.536	−0.535
		*p*	0.610	0.289	0.342	*0.068*	**0.039**	**0.040**
		*p_FDR_*	0.610	0.410	0.410	0.136	0.120	0.120
	Right Parietal CEN ROI	*r*	−0.113	0.401	0.298	−0.179	−0.479	−0.357
		*p*	0.689	0.138	0.281	0.524	0.071	0.192
		*p_FDR_*	0.689	0.384	0.421	0.629	0.384	0.384
	SMN ROI	*r*	−0.045	−0.372	−0.328	−0.034	0.172	0.080
		*p*	0.875	0.172	0.232	0.904	0.539	0.776
		*p_FDR_*	0.904	0.696	0.696	0.904	0.904	0.904
	VN ROI	*r*	−0.055	0.022	−0.012	0.235	−0.062	0.078
		*p*	0.845	0.939	0.967	0.400	0.826	0.782
		*p_FDR_*	0.967	0.967	0.967	0.967	0.967	0.967

ROI, region of interest; DMN, default mode network; SN, salience network; CEN, central executive network; SMN, sensorimotor network; VN, visual network; V_from_N, verb from noun; N_from_V, noun from verb; W_production, overall word production; lnRT, logarithmic transformation of response time; FDR, false discovery rate correction.

## Data Availability

The data presented in this study are available on request from the corresponding author due to ethical reasons.

## Supplementary Materials

The following supporting information can be downloaded at: https://www.mdpi.com/article/10.3390/brainsci14090913/s1, Table S1: Neuropsychological data of HC (healthy control) and PD (Parkinson’s disease) groups of participants.
