# Supplementary material for: The Contribution of Cognitive Control Networks in Word Selection Processing in Parkinson’s Disease: Novel Insights from a Functional Connectivity Study
Source: Brain Sci. 2024 Sep 11;14(9):913. doi: 10.3390/brainsci14090913 (PMC11430391; doi:10.3390/brainsci14090913)
Supplement: Supplementary file 1 [file brainsci-14-00913-s001.zip › brainsci-3163421-supplementary.pdf]

Supplementary Material

The contribution of cognitive control networks in word selection processing in Parkinson's Disease: novel insights from a functional connectivity study

Sonia Di Tella [1,*], Matteo De Marco [2], Isabella Anzuino [1], Davide Quaranta [3,1,4], Francesca Baglio [5] and Maria Caterina Silveri [1]

[1] Department of Psychology, Catholic University of the Sacred Heart, Milan, Italy; sonia.ditella@unicatt.it; isabella.anzuino@unicatt.it; mariacaterina.silveri@unicatt.it;

[2] Department of Life Sciences, Brunel University London, Uxbridge, United Kingdom; matteo.demarco@brunel.ac.uk

[3] Department of Neuroscience, Catholic University of the Sacred Heart, Rome, Italy; davide.quaranta@unicatt.it

[4] Neurology Unit, Fondazione Policlinico Universitario "A. Gemelli" IRCCS, Rome, Italy;

[5] IRCCS Fondazione Don Carlo Gnocchi ONLUS, Milan, Italy; fbaglio@dongnocchi.it

* Correspondence: sonia.ditella@unicatt.it

Table S1. Neuropsychological data of HC (healthy control) and PD (Parkinson's disease) groups of participants.

Neuropsychological data [Mean ± SD]	HC [n=16]	PD [n=18]	Group comparison
MMSE (0–30) [cut-off ≥ 23.80]	27.95 ± 1.30	27.21 ± 1.81	0.187#
Phonological fluency [cut-off ≥ 17.35]	37.48 ± 6.58	36.12 ± 8.98	0.622#
Semantic fluency [cut-off ≥ 25.00]	45.94 ± 5.31	42.17 ± 9.01	0.153#
TMT part A [cut-off ≤ 93.00]	26.69 ± 16.23	50.22 ± 26.89	**0.002°**
TMT part B [cut-off ≤ 282.00]	56.50 ± 33.62	111.10 ± 104.60	0.084°
TMT part B-A [cut-off ≤ 186.00]	31.31 ± 28.60	65.11 ± 85.54	0.152°
Attentional matrices (0–60) [cut-off ≥ 31.00]		49.03 ± 6.08	
Verbal span forward (0–9) [cut-off ≥ 4.26]		6.07 ± 1.06	
Verbal span backward (0–9) [cut-off ≥ 2.65]		4.78 ± 1.07	
Corsi's test forward (0–9) [cut-off ≥ 3.46]		5.23 ± 0.85	
Corsi's test backward (0–9) [cut-off ≥ 3.08]		4.48 ± 0.95	
Immediate recall of 15 words (0–75) [cut-off ≥ 28.53]		49.94 ± 7.72	
Delayed recall of 15 words (0–15) [cut-off ≥ 4.69]		10.80 ± 3.14	
Rey-Osterrieth figure copy (0–36) [cut-off ≥ 28.88]		29.77 ± 5.41	
Rey-Osterrieth figure recall (0–36) [cut-off ≥ 9.47]		14.48 ± 6.93	
FCSRT IFR (0–36) [cut-off ≥ 19.60]		29.26 ± 3.93	
FCSRT ITR * (0–36) [cut-off ≥ 35.00]		35.56 ± 0.81	
FCSRT DFR (0–12) [cut-off ≥ 6.32]		10.43 ± 1.38	
FCSRT DTR * (0–12) [cut-off ≥ 11.00]		11.94 ± 0.25	
FCSRT ISC (0–1) [cut-off ≥ 0.90]		0.86 ± 0.34	
Raven's Coloured Matrices (0–36) [cut-off ≥ 18.96]		29.90 ± 4.84	
M-WCST * (number of completed categories) [cut-off ≥ 3.00]		5.75 ± 1.48	
M-WCST (number of perseverative errors) [cut-off ≤ 6.40]		3.16 ± 4.41	
Stroop test—time interference effect [cut-off ≤ 36.91]		17.80 ± 12.11	
Stroop test—error interference effect [cut-off ≤ 4.23]		0.52 ± 2.00	
Object oral naming (0–30) [cut-off > 28.00]		28.27 ± 1.34	
Action oral naming (0–28) [cut-off > 26.00]		26.07 ± 1.91	

Mean and Standard Deviation are reported. Cut-off in square brackets. SD, Standard Deviation; MMSE, Mini-Mental State Examination; TMT: Trail Making Test; FCSRT: Free and Cued Selective Reminding Test; IFR: immediate free recall; ITR: immediate total recall; DFR: delayed free recall; DTR: delayed total recall; ISC: cueing sensitivity index; M-WCST: Modified Wisconsin Card Sorting test,; * raw scores, when adjusted scores are not available; # Independent samples *t*-test; ° Mann–Whitney U tests.
